# Photodynamic therapy and nanomedicine: current knowledge, limitations, and applications in oral squamous cell carcinoma: a narrative review

**DOI:** 10.3389/fonc.2025.1619560

**Published:** 2025-06-16

**Authors:** Fariba Esperouz, Mauro Lorusso, Andrea Santarelli, Alfredo De Lillo, Lorenzo Lo Muzio, Domenico Ciavarella, Lucio Lo Russo

**Affiliations:** ^1^ Department of Clinical and Experimental Sciences, University of Foggia, Foggia, Italy; ^2^ Department of Clinic Specialistic and Stomatological Sciences, Polytechnic University of Marche, Ancona, Italy

**Keywords:** nanomedicine, photodynamic therapy, oral squamous cell carcinoma, cancer therapy, head and neck

## Abstract

Oral squamous cell carcinoma is still a global health issue, even if oral cavity is an easily accessible anatomical site, the diagnosis is still delayed. Conventional treatments, like chemotherapy and radiotherapy, are usually employed, but not without complications. These drawbacks have necessitated the need for new therapies, one of these is photodynamic therapy (PDT). Nanotechnologies-enhanced PDT has significantly improved tumor targeting, bioavailability, and light absorption, paving the way for its application in early-stage of oral squamous cell carcinoma (OSCC) and as part of combination therapies for advanced cases. Despite the potential advantages of PDT, such as tumor selectivity, minimization of systemic side effects and repeatability of treatment, some limitations still restrict its clinical application. Despite these challenges, its application in oral squamous cell carcinoma and oral potentially malignant disease is promising, both alone or in combination with other therapies. Addressing such pros and cons of this technique, PDT may then be a possible adjuvant tool in the management of OSCC.

## Introduction

Oral squamous cell carcinoma is a global health issue, affecting around 390,000 people worldwide (1); it represents the most common type of head and neck cancer (HNC) accounting about 90% of all oral cancer cases, with an increasing incidence in recent years (2). Although oral cavity is an easily accessible anatomical site for mucosal inspection, oral squamous cell carcinoma (OSCC) diagnosis is often delayed, and for this reason the mortality rate is still high (3).

Treatment strategies for OSCC depend on the disease stage. In early stages surgical intervention or radiotherapy (RT) are usually employed; a different approach with more advanced stages involves a combination of surgery, RT and chemotherapy (CT) (4).

All of these treatment options have their own adverse effects which may be very significant and sometimes burdened with high morbidity. For example, osteoradionecrosis may be associated with RT. Surgery may cause extensive anatomical defects. CT is often associated with oral mucositis, which manifests as inflammation of the oral mucosa with whitish and/or yellowish patches, ulceration, atrophy of the mucosa, erythema, oedema and bleeding, dysgeusia, infectious diseases and xerostomia associated with loss of glandular function (5); it can be very severe and sometimes requires therapy withdrawal.

These drawbacks are the main reasons for the need of new therapies in order to reduce patient’s discomfort, and increase treatment associated morbidity. Hence, different approaches are being explored: one of these is photodynamic therapy (PDT).

It involves the use of a photosensitive agent that, exposed to a specific wavelength light, generates reactive oxygen species (ROS) with cytotoxic effects capable to inhibit the growth of cancer cells (6).

The noninvasive nature of PDT makes it a promising treatment alternative modality, which has been proposed for treating HNC (7), neoplastic lesions of the gastrointestinal tract, lungs, and skin at both premalignant and malignant stages (6).

Nonetheless, PDT’s broader clinical adoption is constrained by intrinsic limitations, such as suboptimal solubility of photosensitizers (PS) their poor tumor specificity, which may end up with unintended phototoxic effects on healthy tissues (8). Modern advances in nanotechnology offer new strategies to overcome these barriers, enhancing PDT’s precision, efficacy, and safety. The nanotechnology-enhanced PDT is an intriguingly evolving landscape which this review is going to address by highlighting novel designs, mechanisms of action, and potential therapeutic benefits.

## Mechanisms of nanotechnology-enhanced PDT: advantages

The basic paradigm of PDT is combining some key components: a selective PS, activated by specific light wavelengths, inducing production of reactive oxygen species (ROS) that selectively may trigger tumor cell apoptosis or necrosis (9). Within this schema, nanotechnologies applied to PDT, which means the use of nanoparticles with PS capability or loaded with PS, may improve tumor targeting, enhance the bioavailability and stability of PS, enhance light absorption and ROS production. (**Table 1**).

**Table 1:** Characteristics of conventional PDT vs. nanotechnology-enhanced PDT.

Feature	Conventional PDT	Nanotechnology-Enhanced PDT
Photosensitizer delivery	Non-specific, systemic	Targeted, via nanocarriers
Tumor selectivity	Limited	Improved via active/passive targeting
Photostability	Often low	Enhanced with nanomaterials
Tissue penetration	Superficial	Potentially deeper (with upconversion NPs)
Side effects	Higher risk (off-target damage)	Reduced due to targeted delivery
Immune activation	Variable	Enhanced (e.g., immuno-PDT)

## Tumor targeting

Tumor targeting can be enhanced with either passive or active mechanisms. Passive targeting exploits the enhanced permeability and retention (EPR) effect, which refers to the tendency of nanoparticles and macromolecules to accumulate in tumor tissues due to the leaky vasculature and poor lymphatic drainage typical of tumor-associated vasculature (10); tumors exhibit abnormal vascularization with permeable blood vessels and inefficient lymphatic drainage, allowing photosensitizer-laden nanoparticles to selectively accumulate into the tumor mass (11).

Active targeting requires ligand-functionalized nanocarriers designed to specifically bind to overexpressed tumor receptors and deliver the photosensitizers (PS) for enhanced photodynamic therapy efficacy (12). Folate-conjugated nanoparticles, Transferrin-conjugated nanoparticles and Antibody-conjugated nanoparticles enables selective accumulation of photosensitizers (PS) in tumor tissues through the mechanism of receptor-mediated endocytosis (13). This allows for a sustained and target release of photosensitizers (PS) (14); with a reduction in drawbacks for the patients, and compliance improvement (9).

## PS bioavailability enhancement

Enhance the bioavailability and stability of PS is another strategy fulfilled by using nanotechnologies. It is based on the utilization of nanocarrier-based delivery systems, including liposomes, dendrimers and polymer-based nanoparticles (PBNPs). These delivery systems have been shown to encapsulate PS molecules, thereby protecting them from premature degradation and extending their systemic circulation (15). By facilitating controlled release, these systems ensure greatest therapeutic impact at tumor sites and minimize systemic toxicity (16).

Liposomal carriers enhance the bioavailability and tumor selectivity of hydrophobic PS. Clinical studies have validated their biocompatibility, reduced immunogenicity, and ability to improve PDT’s therapeutic window for OSCCS, cervical cancer and lung cancer (16).

Polymeric nanoparticles (PNPs), such as those constructed from Poly(lactic-co-glycolic acid)(PLGA) and Polyethylene Glycol (PEG), facilitate sustained PS release while offering significant design flexibility for functionalization. They have been used to merge the above reported strategies (i.e. tumor targeting and PS bioavailability enhancement). In fact, ligand-modified polymeric systems have achieved targeted delivery in preclinical head and neck squamous cell carcinoma studies, highlighting their potential for clinical application (17).PLGA and PEG play a crucial role in nanomedicine by enhancing stability, targeting, and controlled release of photosensitizers in PDT, ultimately increasing therapeutic efficacy while reducing side effects.

## Light absorption and ROS production enhancement

The utilization of nanoparticles (NPs), including but not limited to gold NPs, quantum dots and up conversion NPs (UCNPs), has been demonstrated to enhance light absorption and ROS production (18).These materials exhibit distinguishing optical properties, such as surface plasmon resonance and up conversion luminescence, which enable deeper tissue penetration and more effective tumor ablation. Therefore, this augmentation of PDT’s photodynamic effects frequently results in higher therapeutic outcomes. Metallic nanoparticles amplify light absorption and enhance ROS production through surface plasmon resonance (19); moreover, gold nanorods (GNRs), are preferentially taken up by malignant cells using specific ligands for targeted delivery; indeed, their reflectivity makes possible to distinguish normal tissue from tumors (20).

With a similar mechanism of action, there are quantum dots (QDs) and up conversion nanoparticles, which enable deep tissue PDT by converting low-energy near-infrared light into high-energy emissions that activate PS. In addition, QDs offer intrinsic fluorescence for tumor imaging, enabling real-time monitoring of therapeutic progress (21).

## PDT limitations in clinical applications

Despite the potential advantages of PDT, such as tumor selectivity, minimization of systemic side effects and repeatability of treatment, some limitations still restrict its clinical application.

One of the main problems is that light cannot penetrate deeply into the tumor; usually, it may reach less than 1 cm (22). This problem is of particular significance in the context of deep or bulk tumor masses; in such instances the specific treatment modality of PDT can be suitable only for superficial tumors or the superficial part of tumors, although UCNPs may overcome this limitation due to the fact that these devices uses near-infrared light (23). A further limitation is that PDT relies on the generation of reactive oxygen species (ROS) to induce cell death; solid tumors often have hypoxia, which may significantly reduce the effectiveness of this type of therapy (24).

It is also important to note that patients undergoing PDT may develop hypersensitivity to sunlight and artificial light for days or weeks after treatment, due to the persistence of PS in the skin. This has the potential to impair the patient’s quality of life after treatment (25), and requires to be accurately addressed by future research. In addition, many PS tend to accumulate in healthy tissues, causing side effects and reducing therapeutic efficacy (26). Healthy tissues can be also injured as a consequence of the difficulty to precisely control the depth, intensity and therapeutic effect of PS (26). On the other hand, PDT with nanoparticles may also have the potential, in conjunction with anti-tumor medications (for example CT), to reduce drug toxicity to healthy tissues (27) by increasing treatment selectivity for dysplastic/neoplastic cells.

## PDT and oral squamous cell carcinoma

Photodynamic therapy (PDT) is progressively recognized as a promising alternative treatment for oral squamous cell carcinoma, in particular in early stages tumor, or in adjunct for advantaged cases.

PDT uses a photosensitizing agent that, accumulated into cancer cells, generates reactive oxygen species (ROS) when activated by light.

This type of approach is noninvasive, and more accurate than standard therapies, with a reduction of collateral effects to healthy tissue (28, 29).Recent advances in PDT include the use of nanotechnology to improve the delivery and efficacy of photosensitizers. Nanomaterials such as nanoparticles, liposomes and dendrimers can contain photosensitizers, improving their water solubility, stability and ability to target tumors (27).

These nano-based system also overcome the limitations of conventional PDT, such as insufficient light penetration into deeper tissues and poor solubility of photosensitizers. Nanotechnology allows for enhanced accumulation of PS at the tumor site and better control of treatment depth (6–28).Moreover, PDT has shown efficacy in the treatment of oral leukoplakia and other premalignant lesions by targeting abnormal cells before they progress to invasive cancer (27). In order to reduce side effects, such as mucositis and xerostomia, PDT can be used in combination with other therapies in more advanced stages.

PDT is currently more suitable for superficial lesions and ongoing research is focused on overcoming its limitations in treating deep or metastatic tumors (6–30).

## Conclusions

Photodynamic therapy (PDT) has emerged as a promising alternative treatment for oral squamous cell carcinoma (OSCC), particularly in its early stages or as part of combination therapy for advanced cases. PDT might be effective for superficial lesions, whereas further research should focus on overcoming its limitations for treating deeper or metastatic tumors, as well as in addressing drawbacks related to suboptimal solubility of PS while improving tumor specificity.

PDT offers a more precise treatment approach, but its clinical adoption is still limited by drawbacks: suboptimal solubility of PS, poor and limited light penetration into deeper tissues. The incorporation of nanotechnology is expected to improve the precision, efficacy and safety of PDT, which may then be a possible adjuvant tool in the management of OSCC, especially in the context of integrated treatment modalities including chemotherapy and/or radiotherapy.

## References

[1] Siegel RL Giaquinto AN Jemal A. Cancer statistics, 2024. CA Cancer J Clin. (2024) 74:12–49. doi: 10.3322/caac.21820 38230766

[2] Esperouz F Ciavarella D Lorusso M Santarelli A Lo Muzio L Campisi G. Critical review of OCT in clinical practice for the assessment of oral lesions. Front Oncol. (2025) 15:159197. doi: 10.3389/fonc.2025.1569197 PMC12095244 40406268

[3] Esperouz F Caponio VCA Santarelli A Ballini A Lo Muzio L Ciavarella D. Are we ready to use ultrasounds in the clinical assessment of depth of invasion and tumor thickness in oral squamous cell carcinoma? Results from a systematic review, meta-analysis and trial sequential analysis. Oral Oncol. (2024) 159:107104. doi: 10.1016/j.oraloncology.2024.107104 39551011

[4] Anderson G Ebadi M Vo K Novak J Govindarajan A Amini A. An updated review on head and neck cancer treatment with radiation therapy. Cancers (Basel). (2021) 13:4912. doi: 10.3390/cancers13194912 34638398 PMC8508236

[5] Wen S Brito L Santander J Conteras G. Update on the treatment of chemotherapy and radiotherapy-induced buccal mucositis: a systematic review. Acta Odontol Latinoam. (2023) 36:3–14. doi: 10.54589/aol.36/1/3 37314054 PMC10283397

[6] Mosaddad SA Mahootchi P Rastegar Z Abbasi B Alam M Abbasi K. Photodynamic therapy in oral cancer: A narrative review. Photobiomodul Photomed Laser Surg. (2023) 41:248–64. doi: 10.1089/photob.2023.0030 37222762

[7] Kwiatkowski S Knap B Przystupski D Saczko J Kędzierska E Knap-Czop K. Photodynamic therapy - mechanisms, photosensitizers and combinations. BioMed Pharmacother. (2018) 106:1098–107. doi: 10.1016/j.biopha.2018.07.049 30119176

[8] Zhao W Wang L Zhang M Liu Z Wu C Pan X. Photodynamic therapy for cancer: mechanisms, photosensitizers, nanocarriers, and clinical studies. MedComm. (2020) 2024:5:e603. doi: 10.1002/mco2.603 PMC11193138 38911063

[9] Yan R Liu J Dong Z Peng Q. Nanomaterials-mediated photodynamic therapy and its applications in treating oral diseases. Biomater Adv. (2023) 144:213218. doi: 10.1016/j.bioadv.2022.213218 36436431

[10] Shinde VR Revi N Murugappan S Singh SP Rengan AK. Enhanced permeability and retention effect: A key facilitator for solid tumor targeting by nanoparticles. Photodiagnosis Photodyn Ther. (2022) 39:102915. doi: 10.1016/j.pdpdt.2022.102915 35597441

[11] Izci M Maksoudian C Manshian BB Soenen SJ. The use of alternative strategies for enhanced nanoparticle delivery to solid tumors. Chem Rev. (2021) 121:1746–803. doi: 10.1021/acs.chemrev.0c00779 PMC7883342 33445874

[12] Din FU Aman W Ullah I Qureshi OS Mustapha O Shafique S. Effective use of nanocarriers as drug delivery systems for the treatment of selected tumors. Int J Nanomed. (2017) 12:7291–309. doi: 10.2147/IJN.S146315 PMC5634382 29042776

[13] Subhan MA Yalamarty SSK Filipczak N Parveen F Torchilin VP. Recent advances in tumor targeting via EPR effect for cancer treatment. J Pers Med. (2021) 11:571. doi: 10.3390/jpm11060571 34207137 PMC8234032

[14] Zhuo Y Zhao YG Zhang Y. Enhancing drug solubility, bioavailability, and targeted therapeutic applications through magnetic nanoparticles. Molecules. (2024) 29:4854. doi: 10.3390/molecules29204854 39459222 PMC11510236

[15] Irimie AI Sonea L Jurj A. Future trends and emerging issues for nanodelivery systems in oral and oropharyngeal cancer. Int J Nanomed. (2017) 12:4593–606. doi: 10.2147/IJN.S133219 PMC5500515 28721037

[16] Düzgüneş N Piskorz J Skupin-Mrugalska P Goslinski T Mielcarek J Konopka K. Photodynamic therapy of cancer with liposomal photosensitizers. Ther Deliv. (2018) 9:823–32. doi: 10.4155/tde-2018-0050 30444459

[17] Lee YEK Kopelman R. Polymeric nanoparticles for photodynamic therapy. Biomed Nanotechnol Methods Mol Biol. (2011) 726. doi: 10.1007/978-1-61779-052-2_11 21424449

[18] Guo Q Tang Y Wang S Xia X. Applications and enhancement strategies of ROS-based non-invasive therapies in cancer treatment. Redox Biol. (2025) 18:2025103515. doi: 10.1016/j.redox.2025.103515 PMC11847112 39904189

[19] Serna-Gallén P Mužina K. Metallic nanoparticles at the forefront of research: Novel trends in catalysis and plasmonics. Nano Mater Sci. (2024) 12:20240. doi: 10.1016/j.nanoms.2024.10.009

[20] Hirshberg A Allon I Novikov I Ankri R Ashkenazy A Fixler D. Gold nanorods reflectance discriminate benign from Malignant oral lesions. Nanomedicine. (2017) 13:1333–9. doi: 10.1016/j.nano.2017.01.003 28115253

[21] Li Y Wang Y Shang H Wu J. Graphene quantum dots modified upconversion nanoparticles for photodynamic therapy. Int J Mol Sci. (2022) 23:12558. doi: 10.3390/ijms232012558 36293415 PMC9604409

[22] Al-Jamal AN Al-Hussainy AF Mohammed BA Abbas HH Kadhim IM Ward ZH. Photodynamic Therapy (PDT) in Drug Delivery: Nano-innovations enhancing treatment outcomes. Health Sci Rev. (2025) 14, 2025100218. doi: 10.1016/j.hsr.2025.100218

[23] Sun B Bte Rahmat JN Zhang Y. Advanced techniques for performing photodynamic therapy in deep-seated tissues. Biomaterials. (2022) 291:121875. doi: 10.1016/j.biomaterials.2022.121875 36335717

[24] Ming L Cheng K Chen Y Yang R Chen D. Enhancement of tumor lethality of ROS in photodynamic therapy. Cancer Med. (2021) 10:257–68. doi: 10.1002/cam4.v10.1 PMC7826450 33141513

[25] van Straten D Mashayekhi V de Bruijn HS Oliveira S Robinson DJ. Oncologic photodynamic therapy: basic principles, current clinical status and future directions. Cancers (Basel). (2017) 9:19. doi: 10.3390/cancers9020019 28218708 PMC5332942

[26] Kruger CA Abrahamse H. Utilisation of targeted nanoparticle photosensitiser drug delivery systems for the enhancement of photodynamic therapy. Molecules. (2018) 23:2628. doi: 10.3390/molecules23102628 30322132 PMC6222717

[27] Angjelova A Jovanova E Polizzi A Santonocito S Lo Giudice A Isola G. The potential of nano-based photodynamic treatment as a therapy against oral leukoplakia: A narrative review. J Clin Med. (2023) 12(21):6819. doi: 10.3390/jcm12216819 37959284 PMC10649116

[28] Chen XJ Zhang XQ Liu Q Zhang J Zhou G. Nanotechnology: a promising method for oral cancer detection and diagnosis. J Nanobiotechnol. (2018) 16:52. doi: 10.1186/s12951-018-0378-6 PMC5994839 29890977

[29] Virupakshappa B. Applications of nanomedicine in oral cancer. Oral Health Dent Manage. (2012) 11:62–8. 22692272

[30] Zhao Z Li D Wu Z Wang Q Ma Z Zhang C. Research progress and prospect of nanoplatforms for treatment of oral cancer. Front Pharmacol. (2020) 11:616101. doi: 10.3389/fphar.2020.616101 33391000 PMC7773899

